# Spatial Profiles of Intratumoral PD-1^+^ Helper T Cells Predict Prognosis in Head and Neck Squamous Cell Carcinoma

**DOI:** 10.3389/fimmu.2021.769534

**Published:** 2021-10-28

**Authors:** Kanako Yoshimura, Takahiro Tsujikawa, Junichi Mitsuda, Hiroshi Ogi, Sumiyo Saburi, Gaku Ohmura, Akihito Arai, Saya Shibata, Guillaume Thibault, Young Hwan Chang, Daniel R. Clayburgh, Satoru Yasukawa, Aya Miyagawa-Hayashino, Eiichi Konishi, Kyoko Itoh, Lisa M. Coussens, Shigeru Hirano

**Affiliations:** ^1^ Department of Otolaryngology–Head and Neck Surgery, Kyoto Prefectural University of Medicine, Kyoto, Japan; ^2^ Department of Cell, Developmental & Cancer Biology, Oregon Health and Science University, Portland, OR, United States; ^3^ Department of Pathology and Applied Neurobiology, Kyoto Prefectural University of Medicine, Kyoto, Japan; ^4^ SCREEN Holdings Co., Ltd., Kyoto, Japan; ^5^ Department of Biomedical Engineering, Oregon Health and Science University, Portland, OR, United States; ^6^ Department of Computational Biology, Oregon Health and Science University, Portland, OR, United States; ^7^ Knight Cancer Institute, Oregon Health and Science University, Portland, OR, United States; ^8^ Department of Otolaryngology–Head and Neck Surgery, Oregon Health and Science University, Portland, OR, United States; ^9^ Department of Surgical Pathology, Kyoto Prefectural University of Medicine, Kyoto, Japan; ^10^ Department of Pathology, Japanese Red Cross Kyoto Daini Hospital, Kyoto, Japan

**Keywords:** Head and neck cancer, helper T cell, PD-1, tumor heterogeneity, multiplex immunohistochemistry

## Abstract

**Background:**

Functional interactions between immune cells and neoplastic cells in the tumor immune microenvironment have been actively pursued for both biomarker discovery for patient stratification, as well as therapeutic anti-cancer targets to improve clinical outcomes. Although accumulating evidence indicates that intratumoral infiltration of immune cells has prognostic significance, limited information is available on the spatial infiltration patterns of immune cells within intratumoral regions. This study aimed to understand the intratumoral heterogeneity and spatial distribution of immune cell infiltrates associated with cell phenotypes and prognosis in head and neck squamous cell carcinoma (HNSCC).

**Methods:**

A total of 88 specimens of oropharyngeal squamous cell carcinoma, categorized into discovery (n = 38) and validation cohorts (n = 51), were analyzed for immune contexture by multiplexed immunohistochemistry (IHC) and image cytometry-based quantification. Tissue segmentation was performed according to a mathematical morphological approach using neoplastic cell IHC images to dissect intratumoral regions into tumor cell nests versus intratumoral stroma.

**Results:**

Tissue segmentation revealed heterogeneity in intratumoral T cells, varying from tumor cell nest-polarized to intratumoral stroma-polarized distributions. Leukocyte composition analysis revealed higher ratios of T_H_1/T_H_2 in tumor cell nests with higher percentages of helper T cells, B cells, and CD66b^+^ granulocytes within intratumoral stroma. A discovery and validation approach revealed a high density of programmed death receptor-1 (PD-1)^+^ helper T cells in tumor cell nests as a negative prognostic factor for short overall survival. CD163^+^ tumor-associated macrophages (TAM) provided the strongest correlation with PD-1^+^ helper T cells, and cases with a high density of PD-1^+^ helper T cells and CD163^+^ TAM had a significantly shorter overall survival than other cases.

**Conclusion:**

This study reveals the significance of analyzing intratumoral cell nests and reports that an immune microenvironment with a high density of PD-1^+^ helper T cells in tumoral cell nests is a poor prognostic factor for HNSCC.

## 1 Introduction

Malignant tumor cells interact with various immune cells, tumor-associated stromal cells, secreted molecules and extracellular matrix in the tumor immune microenvironment (TiME), often establishing immunosuppressive environments that support tumor proliferation and metastasis, and to\hat promote immune evasion (1, 2). Based on the expansion of immunosuppressive immune cell lineages, tumor-produced cytokines and chemokines, tumor oncogenes, and mutational landscapes, the immunological architecture of TiME is associated with tumor progression and therapeutic resistance to treatment, providing metrics for developing predictive biomarkers for therapeutic responses (3–5).

Accumulating evidence indicates that intratumoral heterogeneity in the TiME and spatial profiles of immune cell distribution have prognostic significance. The association between the density of tumor-infiltrating lymphocytes and clinical outcomes has been reported in various cancer types, such as ovarian (6), breast (7), pancreas (8, 9) and colorectal cancers (10, 11). Given that intercellular crosstalk occurs in the milieu of multiple cellular components in different proximities, spatial patterns of immune cells can provide a framework for understanding various biological interactions in the TiME, aiding the development of tissue-based biomarkers for predicting clinical outcomes (12). However, a majority of previous studies exploring spatial immune heterogeneity have been limited to comparisons between malignant intratumoral regions and adjacent benign tissues, with a lack of in-depth characterization of tissue components. Focusing on microregional spatial profiles, there is a lack of data on how immune cells are localized inside intratumoral regions, where tumor cell nests and the intratumoral stroma are microscopically mixed in various proportions.

With the advent of immunotherapy, understanding the immune profiles of HNSCC is urgently needed for determining predictive biomarkers to guide therapeutic interventions (13, 14). HNSCC can be divided into human papilloma virus (HPV)-positive and HPV-negative carcinoma, both of which have distinct molecular and immune landscapes. However, intratumor heterogeneity in HNSCC and its prognostic significance is largely unexplored (15, 16). Although the importance of intratumoral helper T cells has been suggested in other cancer types (5, 17), the relationship between CD4^+^ T cells and prognosis is unclear in HNSCC (18). In addition, the spatial profile of helper T cells is unknown.

In this study, based on recent advances in multiplex immunohistochemical (IHC) analyses, enabling *in situ* immune profiling with preserved tissue structure (8), we adopted a tissue segmentation approach for in-depth assessment of intratumoral microregions composed of tumor cell nests and the intratumoral stroma. We aimed to identify the phenotypes and spatial profiles of immune cells in the complex TiME associated with the prognosis of HNSCC.

## 2 Materials and Methods

### 2.1 Discovery Cohort

Data on immune cell densities, spatial information, and clinical characteristics of patients with oropharyngeal squamous cell carcinoma (SCC) (discovery cohort, n = 38) were retrieved from a previous study (8).

### 2.2 Validation Cohort

Formalin-fixed paraffin-embedded (FFPE) samples of oropharyngeal SCC (validation cohort, n = 51, patient characteristics are shown in **Supplementary Table 1**) and benign tonsil samples (controls) were obtained from surgically-resected specimens at Kyoto Prefectural University of Medicine. All tumors were staged according to the 8th edition of the AJCC/UIC TNM classification, and cohort characteristics are shown in **Supplementary Table 1**. HPV-status was determined by p16 staining and/or by quantitative PCR when available.

### 2.3 Multiplex IHC

Multiplex IHC was performed as previously described (8). Briefly, FFPE tumor sections were subjected to sequential immunodetection with validated antibodies identifying discrete leukocyte lineages (**Supplementary Table 2**). Following chromogen development of antibodies, slides were scanned digitally at 20× objective magnification using a NanoZoomer S60 scanner (Hamamatsu Photonics). A complete list of antibodies and conditions used for staining are provided in **Supplementary Table 2**. After staining, image acquisition and computational processing were performed as previously described (8). The three regions of interest (ROI) were identified by an intratumoral high CD3-density area, approximately 6.25 mm^2^ each or less if the analyzable cancerous area was smaller than 3.0 × 6.25 mm^2^. For image preprocessing, coregistration of serially scanned images was performed using Image J/Fiji Version 1.51s (National Institutes of Health) and CellProfiler Version 2.2.0 (Broad Institute). Visualization was performed using Aperio ImageScope Version 12.3.3.5048 (Leica), and Image J. Coregistered images were converted to single-marker images, inverted, and converted to grayscale, followed by pseudo-coloring. For quantitative image assessment, single-cell segmentation and quantification of staining intensity were performed using CellProfiler Version 2.2.0. All pixel intensity and shape-size measurements were saved in a file format compatible with the image cytometry data analysis software FCS Express 7 Image Cytometry (*De Novo* Software).

### 2.4 Tissue Segmentation

Tissue segmentation of tumor cell nests and the intratumoral stroma was performed using an in-house application, IHC Tissue Segmentation Version 1.0, based on images of neoplastic cell IHC. First, the region with tissues defining the ROI and the blank region without tissues were classified based on the maximum thresholding for each image, followed by an image-cleaning algorithm with mathematical morphology operations including opening and closing and a fill hole operation. Second, the tumor cell nest region was calculated by automated thresholding using the Huang fuzzy method performed on the ROI alone, followed by the image-cleaning algorithm described above. Stromal regions were calculated by subtracting the tumor nest regions from the ROI. Finally, a corresponding hematoxylin image was cropped fitting to the result of tissue segmentation, such as tumor cell nests and/or the intratumoral stroma, and analyzed with serially scanned chromogenic images.

### 2.5 Statistics

The Kruskal–Wallis and Wilcoxon signed-rank tests were used to determine statistically significant differences between unpaired and paired data. The Spearman correlation coefficient was used to assess correlations between cell percentages and densities among cell lineages. Overall survival was estimated using the Kaplan–Meier method, and differences were assessed using log-rank tests. Statistical calculations were performed using GraphPad Prism 8.3.0. Cox proportional hazards regression was used to assess the relationship between overall survival and various patient conditions using EZR, which is a graphical user interface for R (The R Foundation for Statistical Computing, Vienna, Austria) (19). Specificity and sensitivity of the density of PD-1^+^ helper T cells were compared using receiver operating characteristic (ROC) curves using EZR. Statistical significance was set at p < 0.05.

## 3 Results

### 3.1 Distribution of Intratumoral T Cells Was Polarized in the Intratumoral Subregions of HNSCC

**Figure 1:**
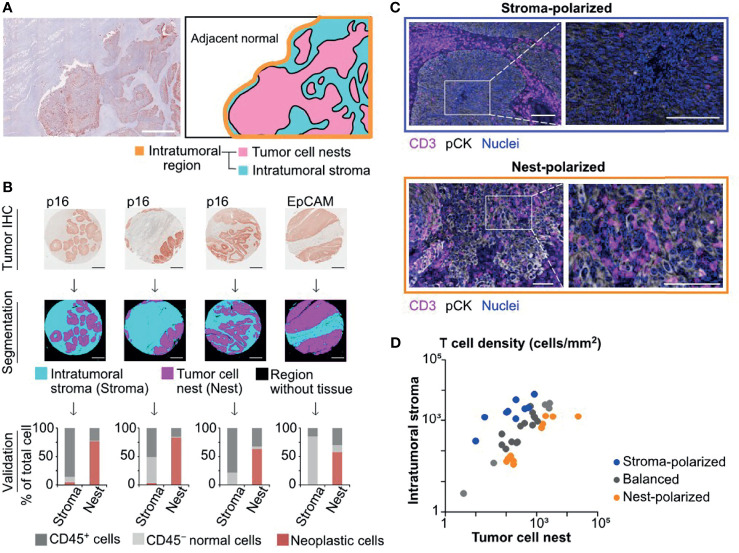
Semi-automated tissue segmentation dissecting intratumoral regions reveals differential degrees of tumor cell nest-infiltration of T cells. **(A)** A superimposed image of hematoxylin and pan-cytokeratin (pCK) staining (left) depicts a representative tissue structure of oropharyngeal head and neck squamous cell carcinoma (SCC). A schematic diagram (right) presents different regions in the intratumoral area that is divided into tumor cell nests and the intratumoral stroma. Scale bar = 500 μm. **(B)** Tumor cell marker IHC images (p16 for HPV-positive, and EpCAM for HPV/p16-negative head and neck squamous cell carcinoma (HNSCC); see *Materials and Methods*) (top) were utilized for semi-automated tissue segmentation classifying into tumor cell nest (Nest), intratumoral stroma (Stroma), and blank regions (middle). Percentages of CD45^+^, CD45^−^ tumor cell marker^−^, and tumor cell marker^+^ cells were analyzed by image cytometry, validating categorization of tumor cells into tumor nest regions (bottom). **(C)** IHC images present differential distributions of T cells (CD3^+^) within tumor cell nests (pCK^+^) and the intratumoral stroma. Scale bars = 100 μm. **(D)** A correlation of T cell densities between the intratumoral stroma and tumor cell nests was shown in cases of oropharyngeal SCC (N = 38). The X and Y axes are shown on a logarithmic scale. Based on the cell density ratios of tumor cell nests to the intratumoral stroma, the polarization status was identified as nest-polarized (>2.0), stroma-polarized (<0.5), and balanced (0.5–2.0).

Since the intratumoral area is microscopically divided into tumor cell nests and the surrounding intratumoral stroma (**Figure 1A**), we developed tissue segmentation algorithms based on tumor cell markers to characterize microregional immune profiles in HNSCC (**Figure 1B**). In this tissue segmentation approach, digitized structural elements of positively stained neoplastic cells were computationally processed by thresholding methods, and each tissue was classified into tumor cell nests versus intratumoral stroma excluding blank regions without tissue (see *Materials and Methods*). Results of tissue segmentation were validated by quantification of cells by multiplex IHC and image cytometry (8), demonstrating that the vast majority of neoplastic cells were categorized into tumor cell nest regions (**Figure 1B** and **Supplementary Figure 1**).

Focusing on the spatial properties of intratumoral T cells, we next assessed the balance of tumor-infiltrating T cells between tumor cell nests and the intratumoral stroma. We observed diversity in the degree of tumor cell nest infiltration by T cells, ranging from “stroma-polarized” cases, in which most of the T cells were trapped in the intratumoral stroma, to “nest-polarized” cases, in which T cells were abundantly infiltrating into the tumor cell nests (**Figure 1C**). While some oropharyngeal SCC cases exhibited a balanced distribution of T cells between tumor cell nests and the intratumoral stroma, most cases exhibited polarity in either direction (**Figure 1D**). These observations indicated that the distribution of “intratumoral” T cells was not necessarily uniform, requiring stratification between tumor cell nests and intratumoral stroma.

### 3.2 Tissue Segmentation Revealed Intratumoral Heterogeneity of Helper T Cell Phenotypes in HNSCC

**Figure 2:**
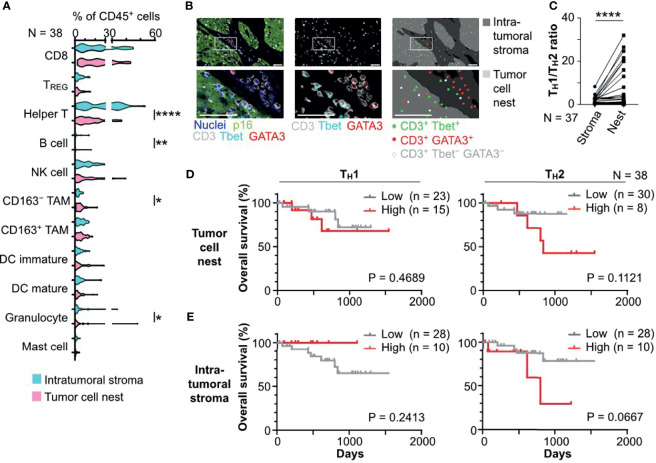
Tissue segmentation elucidates spatially differential phenotypes of immune cell infiltrates in HNSCC. **(A)** Percentages of immune cell lineages of total CD45^+^ cells were shown, comparing the intratumoral stroma and tumor cell nest regions. **(B)** A series of images from an HPV-positive HNSCC tissue section showing differential distribution of Tbet^+^ CD3^+^ (T_H_1) and GATA3^+^ CD3^+^ (T_H_2) cells. Left and middle panels show multiplex IHC images with color annotations. Right panels depict cell identification and location. Boxes represent magnified areas below. Scale bars = 50 μm **(C)** Ratios of T_H_1 to T_H_2, comparing the intratumoral stroma and tumor cell nests, are shown. One case was excluded due to the absence of T_H_2. Statistical differences in **(A)** and **(C)** were determined via Wilcoxon signed rank tests, with *p < 0.05, **p < 0.01, and ****p < 0.0001. **(D, E)** Kaplan–Meier analyses of overall survival of oropharyngeal SCC (N = 38) stratified by cell densities of T_H_1 or T_H_2 infiltrated in tumor cell nests **(D)** and the intratumoral stroma regions **(E)** (cutoff = mean). Statistical significance was determined using the log-rank test.

Based on the tissue segmentation algorithms dissecting the intratumoral regions into tumor cell nests and intratumoral stromal regions, the densities and compositions of 11 different immune cell lineages were quantitatively evaluated by image cytometry (8) (**Figure 2A**). Leukocyte composition analysis based on tissue segmentation revealed significantly higher percentages of helper T cells (CD45^+^CD3^+^CD8^−^Foxp3^−^), B cells (CD45^+^CD3^−^CD20^+^), and CD66b^+^ granulocytes (CD45^+^CD3/CD20/CD56^−^CD66b^+^) in the intratumoral stroma and a higher percentage of tumor-associated macrophages (TAM) (CD45^+^CD3/CD20/CD56^−^CD66b^−^Tyrptase^−^CD68^+^CSF1R^+^), particularly CD163^−^ TAM, in tumor cell nests (**Figure 2A**). Focusing on helper T cells, we observed differential distribution patterns of T_H_1 (CD45^+^CD3^+^CD8^−^Foxp3^−^Tbet^+^) in tumor cell nests and T_H_2 (CD45^+^CD3^+^CD8^−^Foxp3^−^GATA3^+^) in the intratumoral stroma (**Figure 2B** and **Supplementary Figure 2A**). These observations were supported by statistically high ratios of T_H_1/T_H_2 in tumor cell nests (**Figure 2C**), indicating presence of intratumoral heterogeneity in HNSCC. There was no significant impact of tumor cell nest or intratumoral stromal cell densities of T_H_1, T_H_2, CD8^+^ T cells (CD45^+^CD3^+^CD8^+^), and regulatory T cells (T_REG_) (CD45^+^CD3^+^CD8^−^Foxp3^+^) on overall survival (**Figure 2D** and **Supplementary Figures 2B, C**). Taken together, these results indicate that helper T cells, which comprise a large proportion of immune cells, differ greatly in localization and phenotype between tumor cell nests and the intratumoral stroma in HNSCC, indicating that helper T cells deserve a detailed phenotyping approach.

### 3.3 Impact and Spatial Profiles of Intratumoral Programmed Death Receptor-1-Positive T Cells on the Prognosis in HNSCC

**Figure 3:**
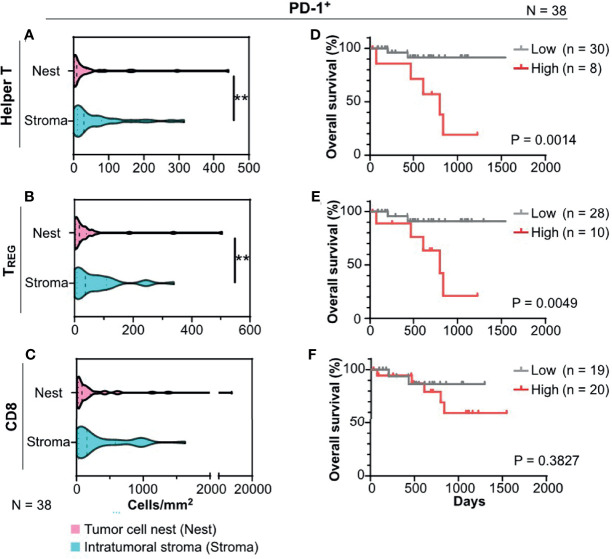
PD-1^+^ T cells infiltrated in tumor cell nests are associated with prognosis of HNSCC. **(A–C)** PD-1^+^ cell densities of helper T cells **(A)**, regulatory T cells (T_REG_) **(B)**, and CD8^+^ T cells (CD8) **(C)** were shown, comparing the intratumoral stroma and tumor cell nests. Statistical differences were determined *via* Wilcoxon signed rank tests, with **p < 0.01. **(D–F)** Kaplan–Meier analyses of overall survival stratified by cell densities of PD-1^+^ cell densities of helper T cells **(D)**, TREG **(E)**, and CD8 **(F)** were shown (cutoff = mean). Statistical significance was determined using the log-rank test.

As the potential impact of programmed death receptor-1 (PD-1) expression on intratumoral T cells has been previously reported (17, 20), we next focused on spatial profiles of PD-1^+^ T cells in HNSCC. Quantification of cell densities with tissue segmentation revealed that tumor cell nests had significantly lower cell densities of PD-1^+^ helper T cells and PD-1^+^ T_REG_ than the intratumoral stroma (**Figures 3A, B**), indicating the presence of spatial heterogeneity in intratumoral PD-1-expressing T cells. Cell densities of PD-1^+^ CD8^+^ T cells did not reveal any statistically significant differences between the tumor cell nests and intratumoral stroma (**Figure 3C**). Notably, a high density of PD-1^+^ helper T cells and PD-1^+^ T_REG_, but not of PD-1^+^ CD8^+^ T cells, in tumor cell nests correlated with short overall survival (**Figures 3D–F**). Similar results were observed even after stratification by HPV status (**Supplementary Figure 3A**). PD-1^+^ T cells, including helper T cells, T_REG_, and CD8^+^ T cells in the intratumoral stroma or the whole cancer tissue region did not correlate with prognosis (**Supplementary Figures 3B, C**), indicating the prognostic significance of cell densities and phenotypes of T cells in tumor cell nests. Together, these data indicate that spatial profiles of PD-1^+^ T cells have a prognostic impact in HNSCC.

### 3.4 PD-1^+^ Helper T Cells in Tumor Cell Nests Were Associated With Short Overall Survival

**Figure 4:**
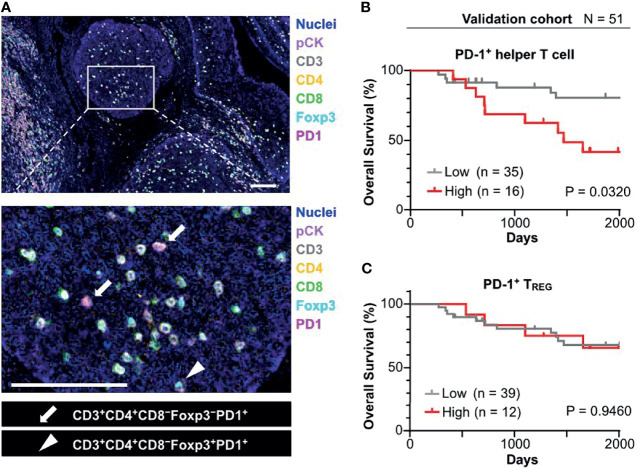
Prognostic significance of PD-1^+^ T cell infiltrated in tumor cell nests in the validation cohort. **(A)** FFPE sections of oropharyngeal SCC (N = 58) were analyzed by a 7-marker multiplex IHC panel. Scale bars = 100 μm. **(B)** Kaplan–Meier analysis of overall survival stratified by density of PD-1^+^ helper T cell in tumor cell nest. The same cutoff value as in the discovery cohort (43.1 cells/mm^2^) was applied. **(C)** Kaplan-Meier analysis of overall survival stratified by density of PD-1^+^ T_REG_ in tumor cell nest (cutoff = mean). Statistical significance in **(B, C)** were determined using the log-rank test.

To validate identified candidates from the discovery cohort, the poor prognostic significance of PD-1^+^ T cells in tumor cell nests was validated by an independent cohort in terms of PD-1^+^ T cell density with tissue segmentation into tumor cell nests and the intratumoral stroma (**Supplementary Table 1**). Since helper T cells are putatively identified by CD3^+^ CD8^−^ populations in the discovery cohort (8), an updated IHC panel including CD4 was applied to the validation cohort to evaluate CD3^+^ CD4^+^ T cell populations as helper T cells (**Figure 4A**). Additionally, instead of p16/EpCAM, pan-cytokeratin (pCK) was used for identification of tumor cell nests to improve versatility. Similar to the findings of the discovery cohort, differential degrees of infiltration of PD-1^+^ helper T cells (CD3^+^CD4^+^) and PD-1^+^ T_REG_ (CD3^+^CD4^+^Foxp3^+^) were observed (**Figure 4A**). When the same cutoff value from the discovery cohort was applied to the validation cohort, a high density of PD-1^+^ helper T cells in tumor cell nests was significantly correlated with short overall survival (**Figure 4B**). The Cox proportional hazards model showed that a high density of PD-1^+^ helper T cells in tumor cell nests as an unfavorable prognostic factor, independent of HPV status, clinical stage, and smoking history (**Table 1**). This tendency was preserved regardless of HPV status, analogous to the discovery cohort (**Supplementary Figures 4B, C**). When these results were examined in the discovery and validation groups with the cutoff optimized by the ROC curve, the same results were obtained (**Supplementary Figures 5A, B**). PD-1^+^ T_REG_ in tumor cell nests did not show prognostic significance, even at the optimal cutoff value (**Figure 4C**). These observations indicate that PD-1^+^ helper T cells in tumor cell nests reflect poor prognostic factors.

**Table 1:** Variables associated with overall survival: Cox proportional hazards model (Validation cohort, N = 51).

Variables	HR	95%CI	*P* value
HPV-negative	4.97	(1.34–18.4)	0.017
Male	1.78	(0.21–15.02)	0.6
Presence of smoking history	0.97	(0.12–7.97)	0.98
Stages III–IV	0.46	(0.13–1.57)	0.22
High PD-1^+^ helper T cell density in tumor cell nests	3.32	(1.15–9.55)	0.026

Detailed clinicopathological information is available in **Supplementary Table 1**.

### 3.5 Negative Prognostic Significance of PD-1^+^ Helper T Cells in Tumor Cell Nests Was Related to Colocalization With CD163^+^ TAM

**Figure 5:**
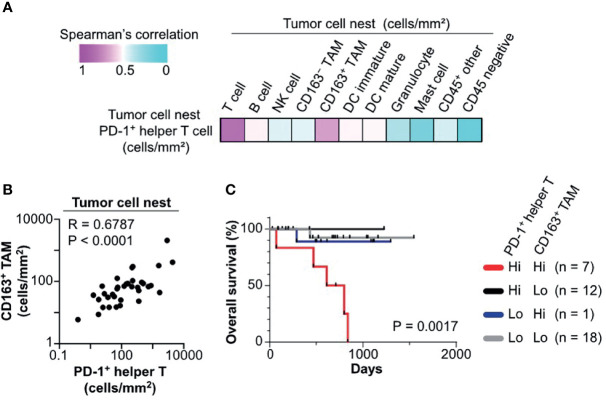
Colocalization of PD-1^+^ helper T cells with CD163^+^ TAM is associated with poor prognosis. **(A)** A heat map according to color scale (upper left) presents spearman correlations of cell densities of PD-1^+^ helper T cells versus various cell lineages within tumor cell nests. **(B)** Spearman correlation coefficient shows a correlation between PD-1^+^ helper T cells and CD163^+^ tumor associated macrophages (TAM) in tumor cell nest regions. The X and Y axes are shown on a logarithmic scale. **(C)** Kaplan–Meier analysis of overall survival stratified by cell densities of PD1^+^ helper T cell (cutoff = mean) and CD163^+^ TAM (cutoff = median) in tumor cell nests. Statistical significance was determined using the log-rank test.

We next explored cell–cell relationships underlying the negative prognostic significance of PD-1^+^ helper T cells in tumor cell nests. Correlation analysis of cell densities of PD-1^+^ helper T cells and various cell lineages in tumor cell nests was performed in the discovery cohort. Notably, PD-1^+^ helper T cells correlated with CD163^+^ TAM and total T cells, but not with CD163^−^ TAM and other lineages (**Figure 5A**). Cell densities of PD-1^+^ helper T cells in tumor cell nests were significantly correlated with those of CD163^+^ TAM in tumor cell nests (R = 0.6787, p < 0.0001; **Figure 5B**). Since CD163^+^ TAM have been reported to promote the immunosuppressive microenvironment and cancer progression (1, 21), we evaluated the potential prognostic impact of PD-1^+^ helper T cells and CD163^+^ TAM in tumor cell nests. A subgroup with high cell densities of both PD-1^+^ helper T cells and CD163^+^ TAM exhibited significantly shorter overall survival than the other subgroups (**Figure 5C**). Together, these observations indicate that tumor cell nest-specific colocalization of PD-1^+^ helper T cells and CD163^+^ TAM reflects a tumor-promoting microenvironment and disease aggressiveness.

## 4 Discussion

In this study, we investigated immune cell localization in HNSCC tumors using multiplex IHC and tissue segmentation, revealing tumor cell nest-based characteristics of immune cell phenotypes and prognostic significance. With the increasing attention focused on single-cell analysis, various cellular components in the TiME have been revisited in terms of spatial relationships and tissue context. In many cancer types, aggregation of tumor cells can form a tumor cell nest-specific microenvironment, which is advantageous for cancer cell survival and progression *via* different metabolic, hypoxic, and inflammatory conditions (22, 23). Although cancer-intrinsic factors are deeply associated with recruitment or exclusion of immune infiltrates, immune characteristics within tumor cell nests are not well understood, mainly due to the lack of single-cell technology with preserved tissue structure. This study provided an in-depth classification of intratumor regions into tumor cell nests and the intratumoral stroma (**Figure 1A**) and demonstrated microregional heterogeneity in relation to disease aggressiveness.

Since immune cells require multiple markers for lineage and phenotype identification, quantitative evaluation of immune cell lineages with spatial information has been technically challenging due to the limited number of analyzable markers in conventional IHC and immunofluorescence methodologies. To tackle those grand challenges, a variety of new approaches have been actively developed in the field of head and neck cancer as well as various malignancies *via* seminal technologies such as NanoString GeoMx digital spatial profiling (24), Vectra Polaris (16), co-detection by indexing (25), Visium spatial gene expression analysis (26) and other mIHC technologies (12, 27, 28). Owing to recent advances in multiplex IHC and immunofluorescence technologies as well as spatial genomics and molecular imaging technologies, we adopted a 12-marker chromogenic multiplex IHC platform with tissue segmentation algorithms, enabling identification of cellular components with preserved tissue architecture (**Figures 1B–D**). Although many of the latest technologies have better resolution and multiplexing abilities, our chromogenic mIHC has the advantages of being 1) inexpensive equivalent to standard IHC, 2) capable of visualizing and quantifying whole tissues, and 3) based on clinically used immunohistochemical methods, making it practical and close to clinical application (8). In this study, the spatial properties of T cells in the intratumoral subregions were quantitatively evaluated in terms of prognostic assessment, suggesting that multiplexed imaging methods can provide a basis for understanding intratumoral heterogeneity and tissue-based biomarker exploration *via* potential combination of current mIHC and other state-of-the-art technologies in the future.

Based on multiplex IHC analyses with tissue segmentation, the major findings of this study were: 1) polarized T_H_1/T_H_2 balance (**Figure 2**); and 2) poor prognostic significance of PD-1^+^ helper T cells in HNSCC tumor cell nests (**Figures 3**, **4**). Despite recent controversies (29–31), helper T cells are considered to function differentially depending on their phenotypes thus have been classically divided into T_H_1 and T_H_2 (32, 33). The T_H_1–T_H_2 paradigm has been largely known to be associated with immune characteristics, where T_H_1 has been traditionally considered a functional phenotype that enhances anti-tumor immunity *via* regulation and maintenance of effector and memory functions of CD8^+^ T cells (34, 35). Simultaneously, a wide range of studies across various types of cancer have revealed that PD-1 on T cells is a major inhibitory receptor that regulates T cell dysfunction, compromising the ability to eliminate antigenic tumor cells (15, 20, 36). In agreement with previous studies indicating that PD-1^+^ T cells is associated with poor prognosis in breast cancer before the era of immunotherapy (37, 38), our data demonstrate that a high density of PD-1^+^ helper T cells in tumor cell nests was a significant poor prognostic factor in HNSCC that was not treated by immunotherapy (**Figures 3**, **4**). Given that PD-1 expression on T cells is induced by continuous exposure to antigens (39), the predominant expression of PD-1 within tumor cell nest-infiltrated T cells, particularly with T_H_1 phenotypes, may be related to the abundance of tumor antigens in tumor cell nests and reduced intrinsic antitumor immunity. Considering that the presence of PD-1^+^ T cells correlates with favorable response to immune checkpoint blockade in melanoma, non-small cell lung cancer, and gastric cancer (17, 20), the high frequency of PD-1^+^ helper T cells in tumor cell nests might be a potential therapeutic target by immune checkpoint blockade.

HNSCC has been known to possess a T_H_2-polorized microenvironment (40, 41), where abundance of CD8 T cells (42), and helper T cells including PD-1^+^ T cells have favorable prognostic significance (42, 43). However, recent single-cell analysis revealed the existence of tumor heterogeneity within head and neck cancer tissues, and the need for detailed analysis of HNSCC, including spatial information (44). In the context of tumor heterogeneity, the present study demonstrated that T_H_1 predominance in the tumor cell nests in HNSCC (**Figure 2**) as well as poor prognostic significance of PD-1^+^ helper T cells in the tumor cell nests (**Figures 3**, **4**). Given that the whole tumor tissue is basically T_H_2-dominant (45) and the frequency of PD-1^+^ helper T cells in the whole tissue is associated with favorable prognosis (43), the present study focusing on tumor cell nests showed the opposite results to the whole tumor. Although T_H_1-polarization within the tumor cell nests, especially in HPV-positive cancers (**Figure 2**), suggests the potential involvement of T-cell immunity in response to tumor-specific antigens, our data simultaneously indicates that T_H_1-polarized helper T cells could be dysfunctional in view of high expression of PD-1 (**Figure 3**), which can serve as one of T cell exhaustion markers such as Eomes, TIM-3, LAG-3, etc (46). Interestingly, in this study, PD-1^+^ helper T cells were found to co-localize with TAM in the tumor cell nests (**Figure 5**), suggesting the presence of microregional immune-microenvironmental profiles associated with immunosuppression, potentially leading to dysfunctional status of helper T cells. Those observations highlight the importance of spatially-resolved understanding of the heterogeneity of immune microenvironment in HNSCC.

To explore the mechanisms underlying the negative prognostic impact of PD-1^+^ helper T cells, we comprehensively investigated the potential relationships between PD-1^+^ helper T cells and various immune cells, indicating potential interactions between PD-1^+^ helper T cells and myeloid cells (**Figure 5**). CD163^+^ TAM, which have similar properties to M2 macrophages, are associated with an immunosuppressive microenvironment and unfavorable clinical outcomes in breast, bladder, ovarian, gastric, and prostate cancers (47–49). Furthermore, several preclinical and clinical studies have indicated a synergic effect of inhibiting TAM and the PD-1/PD-L1 axis (21). Although detailed biological mechanisms need to be explored in future studies, these studies indicate that colocalization of TAM and PD-1^+^ helper T cells can serve as an additional therapeutic target against the immunosuppressive tumor cell nest-specific microenvironment.

One of possible limitations of this study is that the discovery and validation cohorts were derived from archived specimens from the pre-immunotherapy era for HNSCC; thus, potential correlations between PD-1^+^ T cells and response to immunotherapy were not included in this study. Given that intratumoral PD-1^+^ helper T cells correlate with response to immune checkpoint inhibitors in melanoma (17), the observed negative prognostic significance of PD-1^+^ helper T cells by conventional chemotherapy may provide a favorable indication for anti-PD-1 therapy in combination with cytotoxic therapeutics in HNSCC. While PD-1 is considered to be one of T cell exhaustion markers (46), the functional status of PD-1^+^ helper T cells identified in our study was not investigated at this time, and should be verified by future functional analysis. Additionally, although this study focused on the tumor cell nests rather than the tumor-surrounding stroma, the phenotypes and origin of intratumoral stroma is also important, particularly in oropharyngeal cancer. Given that the intratumoral stroma can have heterogenous background such as newly developed by cancer cells, and the remnants of normal structures due to direct invasion from cancer cells, further studies are required for understanding spatial profiles of immune microenvironment focusing on the intratumoral stroma.

In summary, this study demonstrated that dividing the intratumor region into tumor cell nests and the intratumoral stroma provides an in-depth understanding of the microregional characteristics of the TiME. Since a high density of PD-1^+^ T cells in tumor cell nests was identified as a predictor of prognosis, monitoring spatial phenotypes of PD-1^+^ helper T cells may provide a stratification for optimized treatment in HNSCC.

## Data Availability Statement

The original contributions presented in the study are included in the article/**Supplementary Material**. Further inquiries can be directed to the corresponding author.

## Ethics Statement

The studies involving human participants were reviewed and approved by Oregon Health & Science University (protocol #809 and #3609) and the Kyoto Prefectural University of Medicine (ERB-C-43-4). The patients/participants provided their written informed consent to participate in this study.

## Author Contributions

KY designed and performed experiments, analyzed data, and wrote the manuscript. TT conceived and designed the experiments, interpreted the data, and wrote the manuscript. JM and SSa performed experiments. GO, AA, and DC provided patient samples and clinical expertise. HO, SSh, GT, and YC provided computational support for image analysis. SY, AM-H, EK, and KI provided pathological expertise. LC reviewed and edited the manuscript and provided funds and scientific oversight for initial development of the discovery cohort. SH supervised the study. All authors contributed to the article and approved the submitted version.

## Funding

This study was supported by grants from the Japanese Ministry of Education, Culture, Sports, Science and Technology (17H07016, 19K18814, 19K18739, and 19K23893), the Public Promoting Association Asano Foundation for Studies on Medicine, and the Research Promotion Award from the Oto-Rhino-Laryngological Society of Japan, Inc. The funder was not involved in the study design, collection, analysis, interpretation of data, the writing of this article or the decision to submit it for publication.

## Conflict of Interest

TT is a paid consultant for Ono Pharmaceutical and receives speaker fees from Merck Sharp & Dohme Corp, Ono Pharmaceutical, and Bristol-Myers Squibb. HO is an employee of SCREEN Holdings Co., Ltd. SSh is an employee of SCREEN Holdings Co., Ltd. EK is a paid consultant for Roche Diagnostics and receives speaker fees from Chugai Pharmaceutical. KI received research funding from the SCREEN Holdings Co., Ltd. LC is a paid consultant for Cell Signaling Technologies, AbbVie Inc., and Shasqi Inc., received reagent and/or research support from Plexxikon Inc., Pharmacyclics, Inc., Acerta Pharma, LLC, Deciphera Pharmaceuticals, LLC, Genentech, Inc., Roche Glycart AG, Syndax Pharmaceuticals Inc., Innate Pharma, NanoString Technologies, and Cell Signaling Technologies, is a member of the Scientific Advisory Boards of Syndax Pharmaceuticals, Carisma Therapeutics, Zymeworks, Inc, Verseau Therapeutics, Cytomix Therapeutics, Inc., Hibercell, Inc., Alkermes, Inc., Genenta Sciences, and Kineta Inc, and is a member of the Lustgarten Therapeutics Advisory working group, and the AstraZeneca Partner of Choice Network.

The remaining authors declare that the research was conducted in the absence of any commercial or financial relationships that could be construed as a potential conflict of interest.

## Publisher’s Note

All claims expressed in this article are solely those of the authors and do not necessarily represent those of their affiliated organizations, or those of the publisher, the editors and the reviewers. Any product that may be evaluated in this article, or claim that may be made by its manufacturer, is not guaranteed or endorsed by the publisher.

## Supplementary Material

Supplementary Figure 1 Image cytometry-based cell population analyses and gating strategies. **(A)** Gating thresholds for qualitative identification were determined based on each specimen’s comparison. The X and Y axes are shown on a logarithmic scale. **(B)** Location of each cell in reference to image cytometry is visualized by pseudo-colored picture plot and location plot.

Supplementary Figure 2 Multiplex IHC images and survival analysis for regulatory T cells (T_REG_) and CD8^+^ T cells (CD8). **(A)** Single channel images in support of **Figure 3B**. An immunostaining of Nuclei, p16, CD3, Tbet, and GATA3 in an HPV-positive HNSCC tissue section. Scale bars = 50 μm. Boxes represent magnified areas below. **(B, C)** Kaplan–Meier analysis of overall survival stratified by density of T_REG_ **(B)** and CD8 **(C)** in tumor cell nests (cutoff = mean). Statistical significance was determined using the log-rank test.

Supplementary Figure 3 Prognostic significance of T cells in relation to HPV-status and tissue segmentation. **(A)** Kaplan-Meier analysis of overall survival stratified by density of PD-1^+^ helper T cell in the intratumoral stroma of HPV-positive cases (left) and HPV-negative cases (right). **(B, C)** Kaplan–Meier analysis of overall survival stratified by density of PD-1^+^ helper T cells **(B)** and PD-1^+^ T_REG_ **(C)** in the intratumoral stroma and whole cancer tissue. In **(A–C)**, the respective mean value was used as the cutoff, and statistical significance was determined using the log-rank test.

Supplementary Figure 4 7-marker multiplex IHC panel reveals prognostic significance of PD-1^+^ helper T cells. **(A)** Single channel images in support of **Figure 4A**. An immunostaining of nuclei, pCK, CD3, CD4, CD8, Foxp3, PD-1, and CD66b in an HNSCC tissue section. Scale bars = 100 μm. **(B, C)** Kaplan–Meier analysis of overall survival stratified by density of PD-1^+^ helper T cell in the intratumoral stroma of HPV-positive cases **(B)** and HPV-negative cases **(C)** in the validation cohort. The respective mean value was used as the cutoff value, and statistical significance was determined using the log-rank test.

Supplementary Figure 5 Prognostic significance of PD-1^+^ T cells infiltrated in tumor cell nests stratified by receiver operating characteristic (ROC)-derived cut-off. **(A, B)** ROC curves were shown comparing specificity and sensitivity of the density of PD-1^+^ helper T cells infiltrated in the tumor cell nest in the discovery **(A)** and validation **(B)** cohorts. **(C, D)** Kaplan-Meier analysis of overall survival stratified by density of PD-1^+^ helper T cell infiltrated in the tumor cell nest in the discovery **(C)** and validation **(D)** cohorts. Cut-off was determined using ROC analyses in **(A, B)**. Statistical significance was determined using the log-rank test.

Supplementary Table 1 Patient characteristics in the validation cohort.

Supplementary Table 2 A complete list of antibodies and conditions used for staining.

## Abbreviations

TiME, tumor immune microenvironment; HNSCC, head and neck squamous cell carcinoma; HPV, human papilloma virus; IHC, immunohistochemistry; TAM, tumor-associated macrophages; SCC, squamous cell carcinoma; FFPE, formalin-fixed paraffin-embedded; ROI, regions of interest; TREG, regulatory T cells.
