# Supplementary figures and images for: Spatial Profiles of Intratumoral PD-1+ Helper T Cells Predict Prognosis in Head and Neck Squamous Cell Carcinoma
Source: Front Immunol. 2021 Oct 28;12:769534. doi: 10.3389/fimmu.2021.769534 (PMC8581667; doi:10.3389/fimmu.2021.769534)

**Fig. S1****A**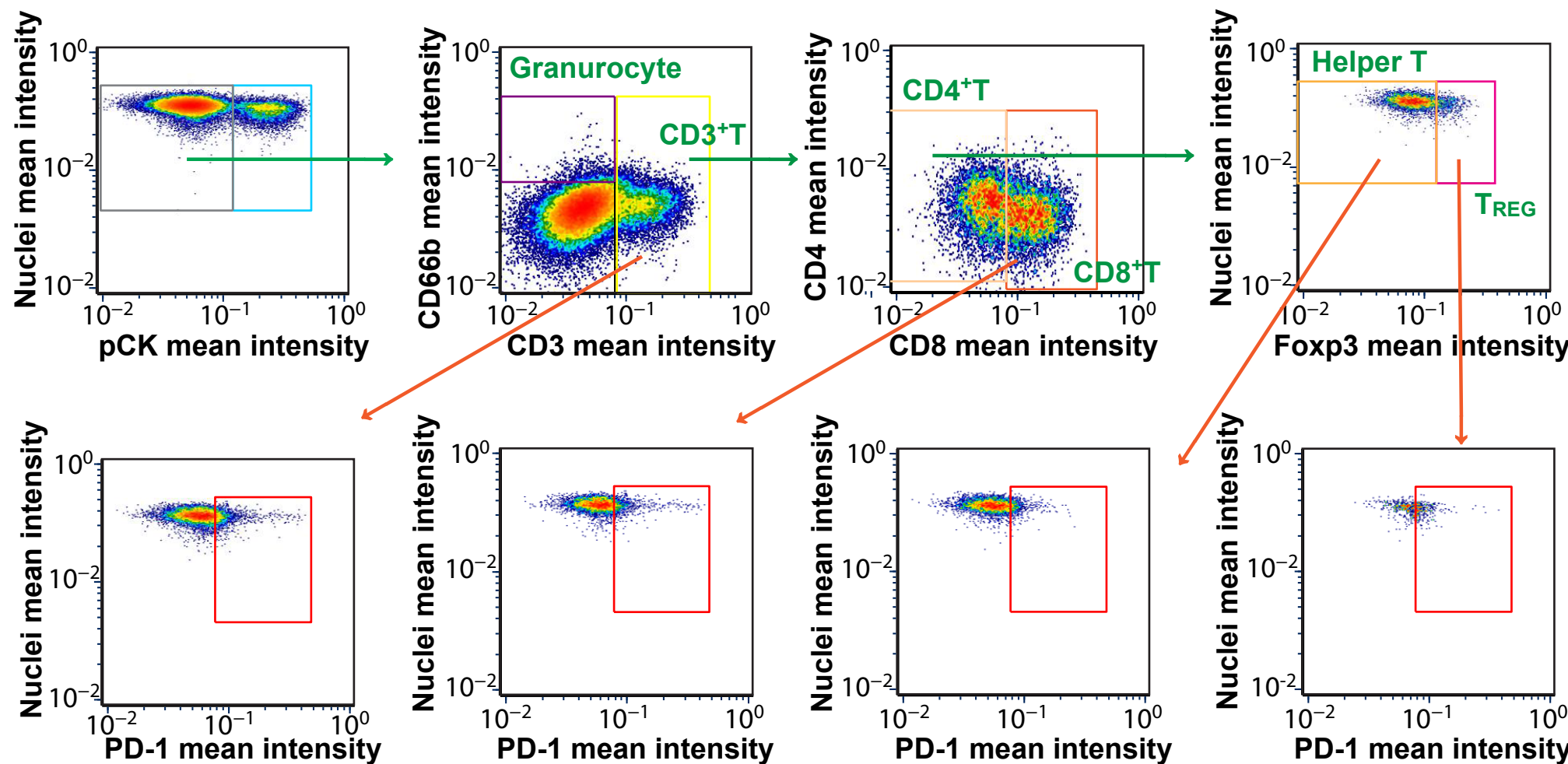**B**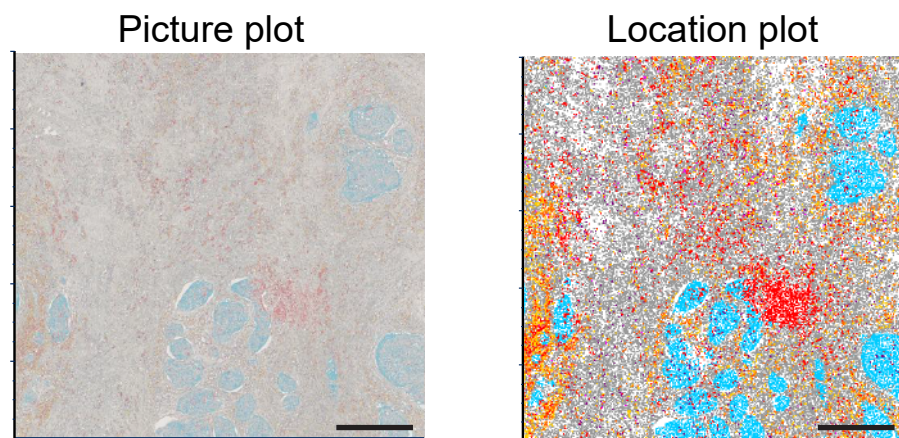

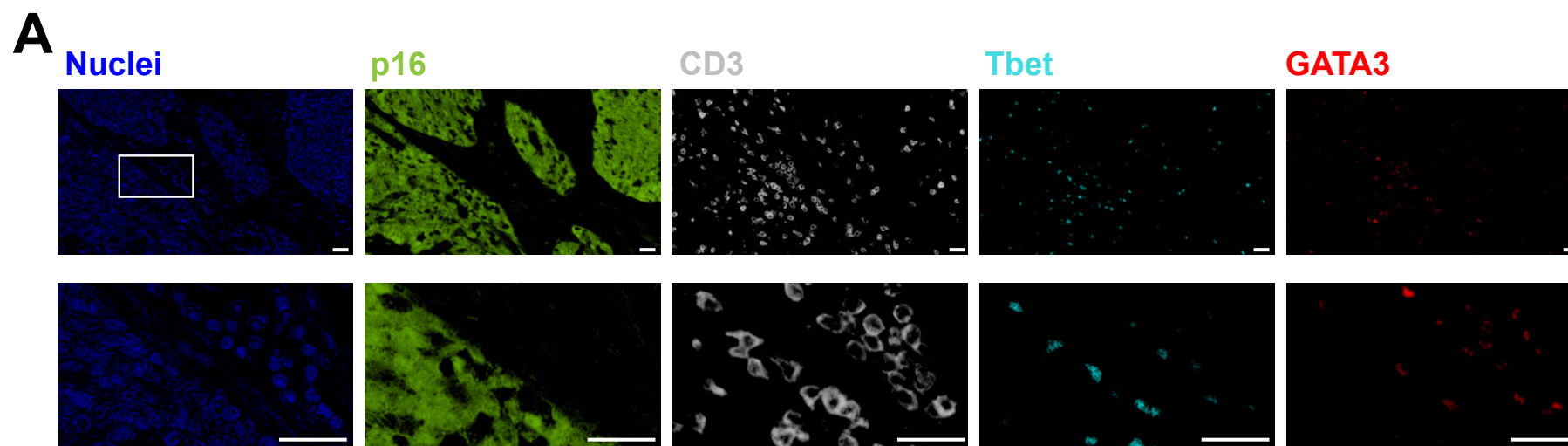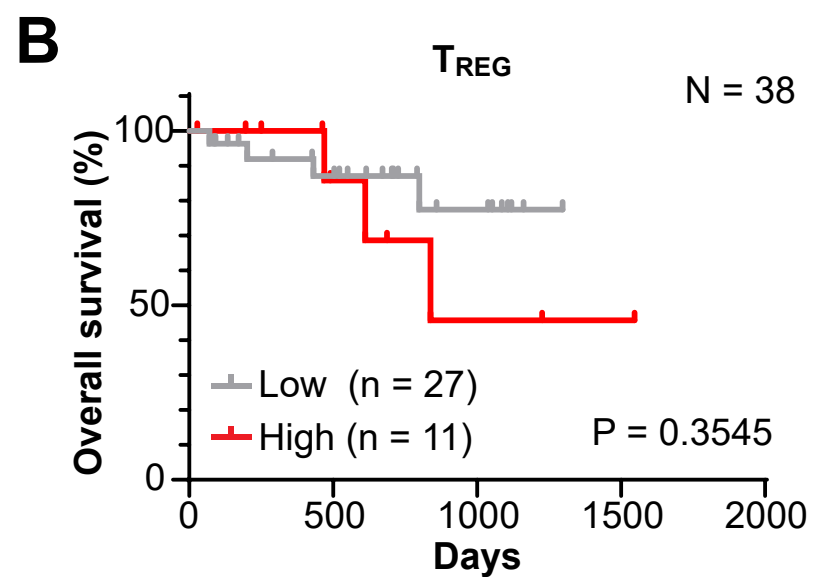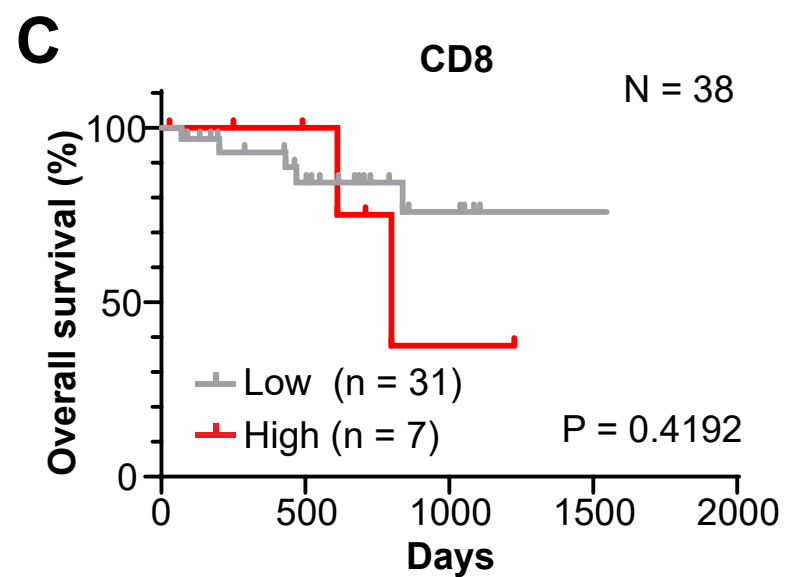

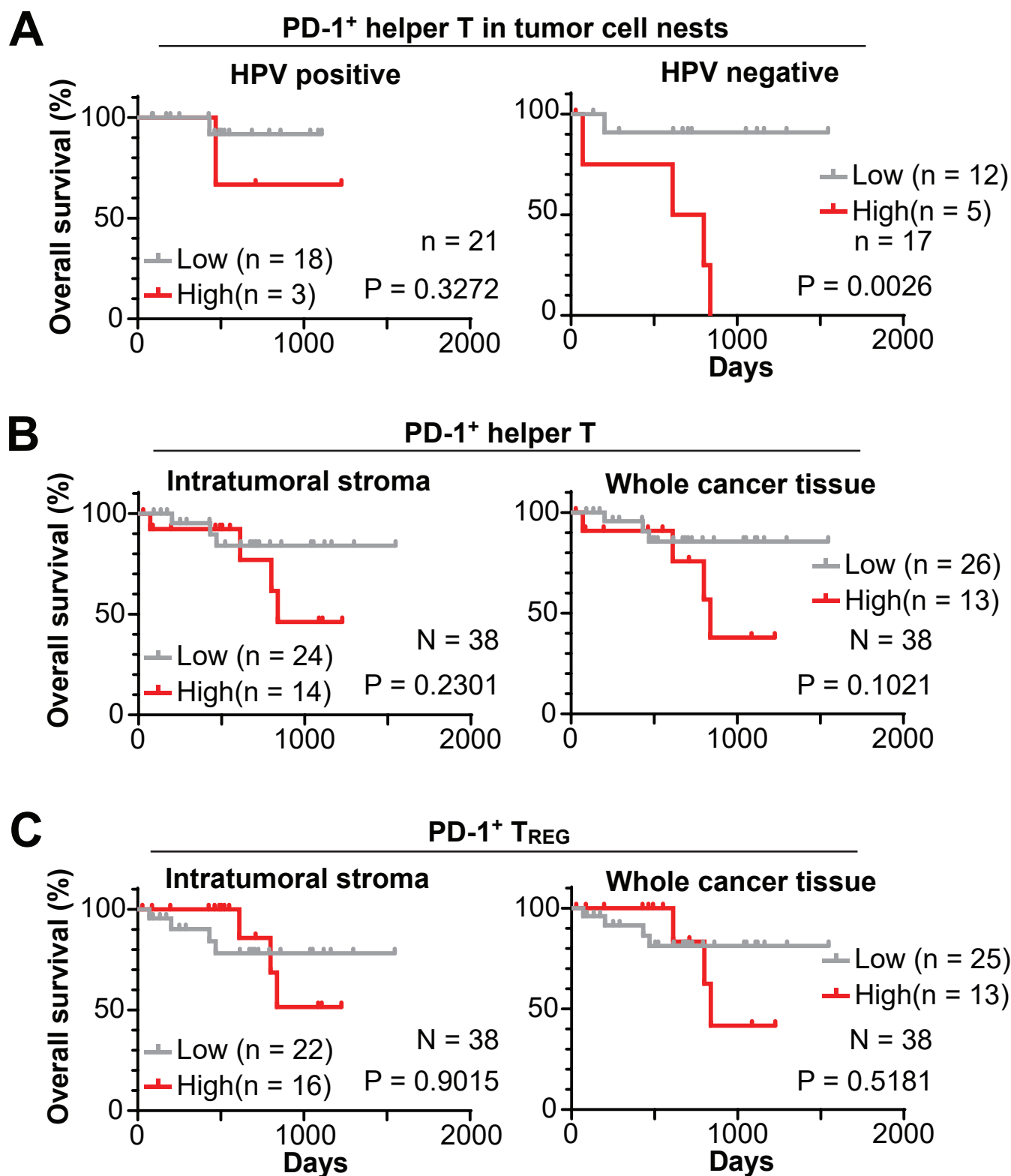

**A**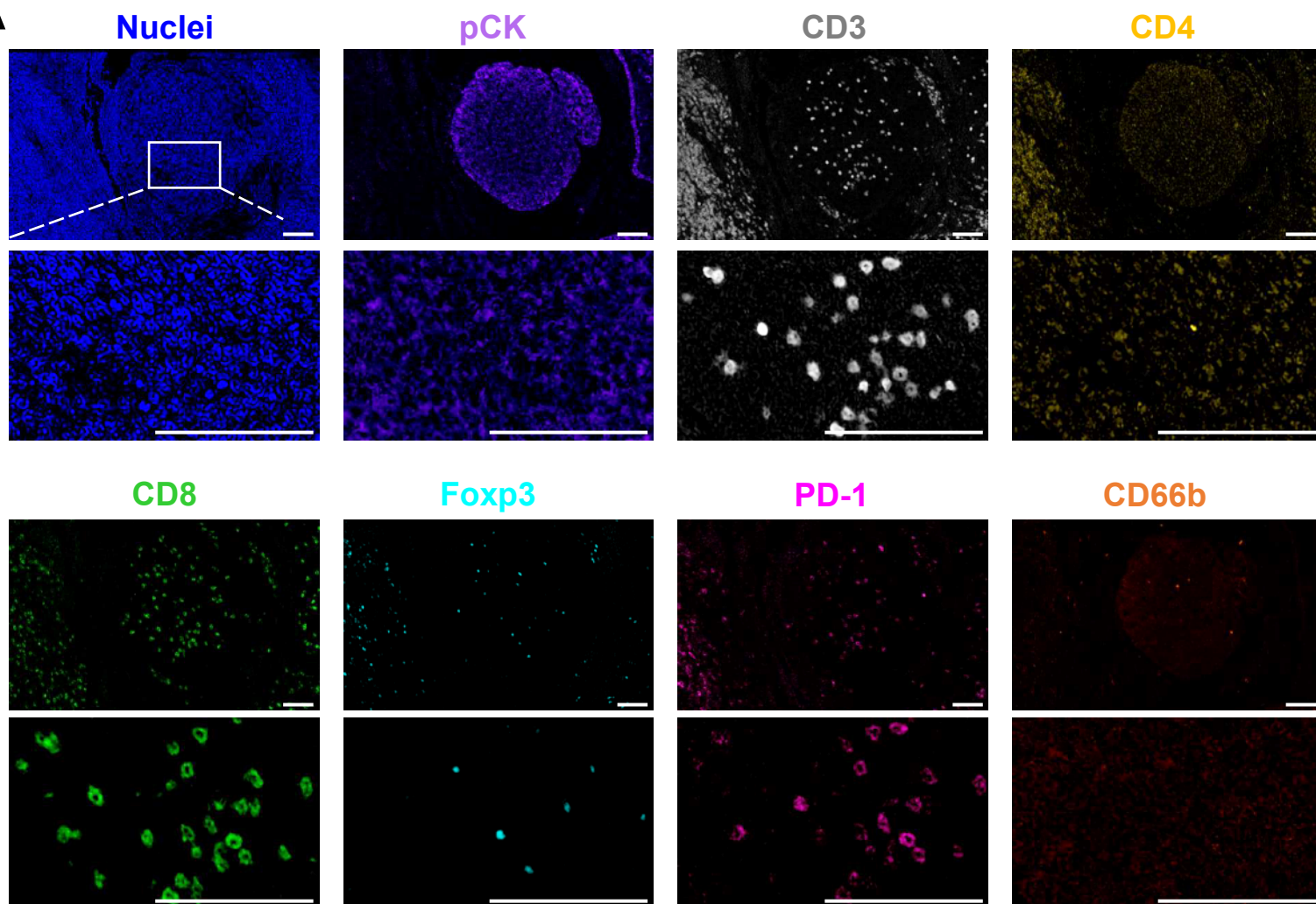**B**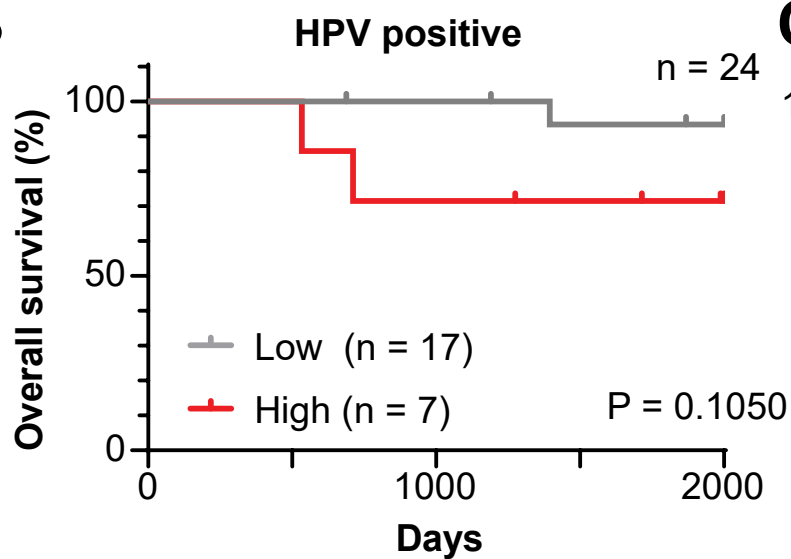**C**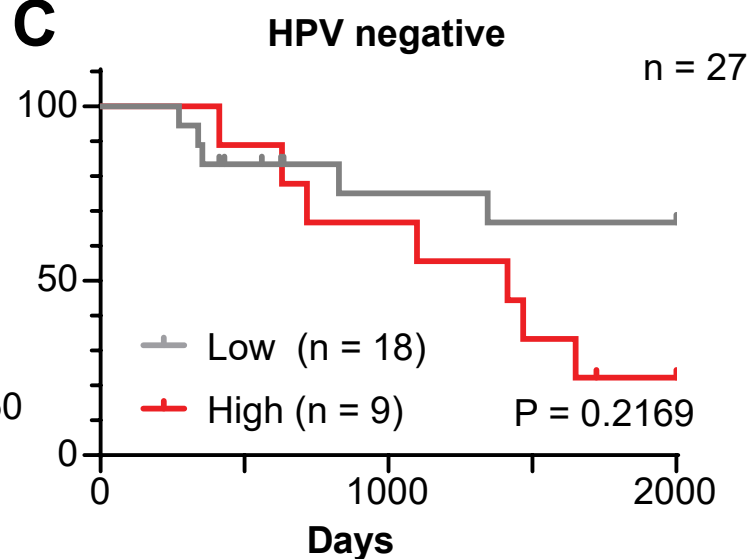

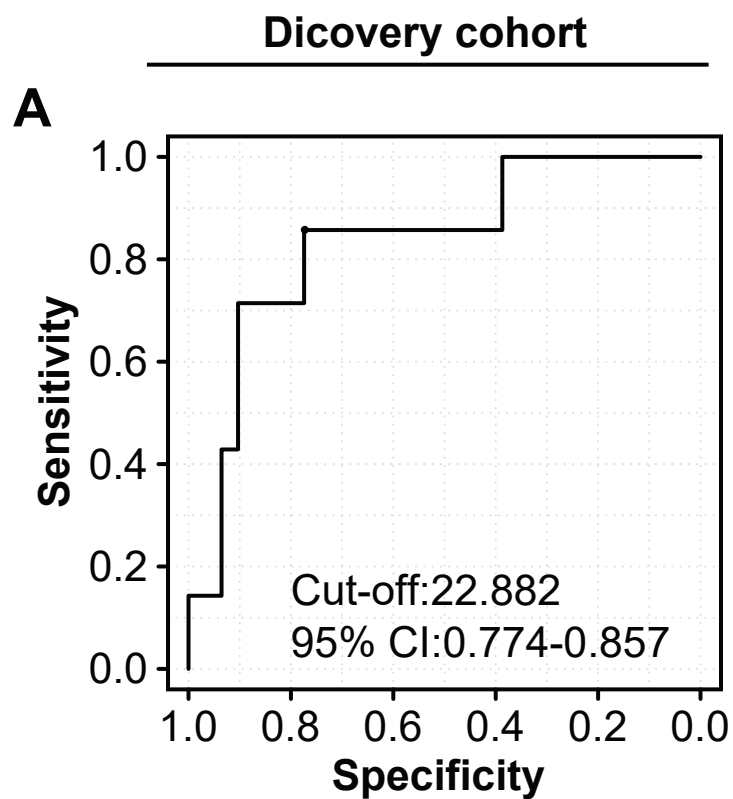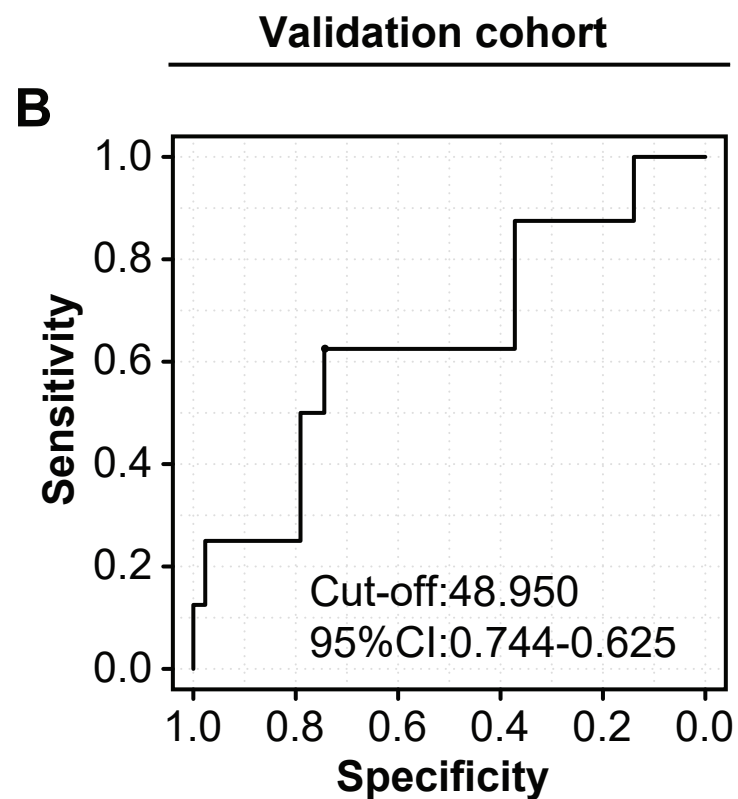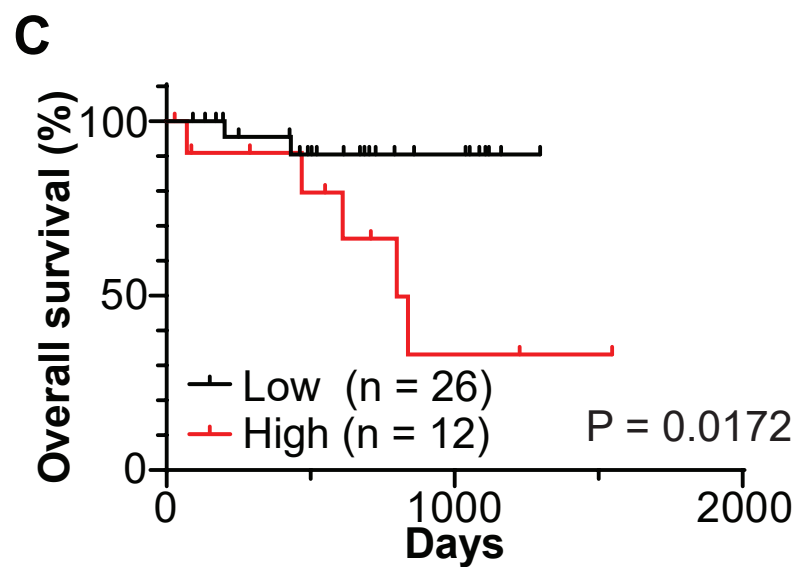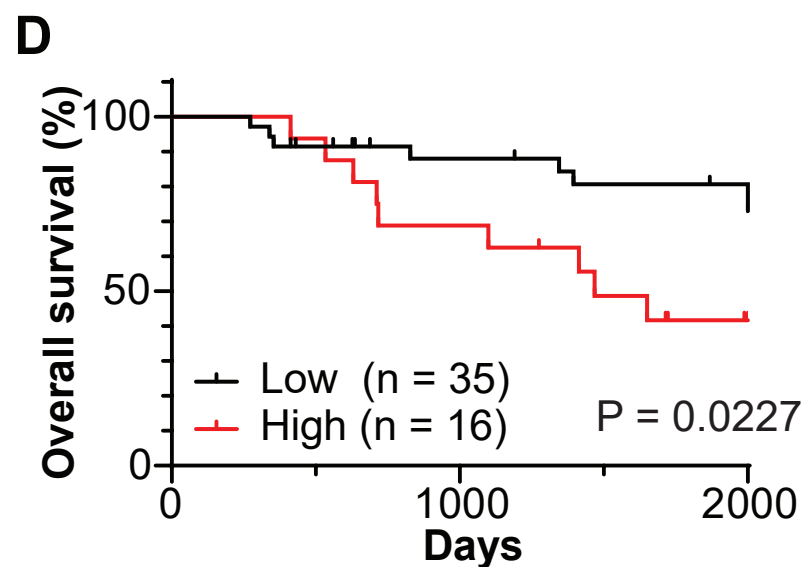

Supplement: Supplementary Figure 1 — Image cytometry-based cell population analyses and gating strategies. (A) Gating thresholds for qualitative identification were determined based on each specimen’s comparison. The X and Y axes are shown on a logarithmic scale. (B) Location of each cell in reference to image cytometry is visualized by pseudo-colored picture plot and location plot. [file DataSheet_1.pdf]
