# Supplementary material for: Spatial Profiles of Intratumoral PD-1+ Helper T Cells Predict Prognosis in Head and Neck Squamous Cell Carcinoma
Source: Front Immunol. 2021 Oct 28;12:769534. doi: 10.3389/fimmu.2021.769534 (PMC8581667; doi:10.3389/fimmu.2021.769534)
Supplement: Supplementary Table 1 — Patient characteristics in the validation cohort. [file Table_1.docx]

**Supplementary Table 1**. Patient characteristics in the validation cohort

| Features |  | All patients | HPV-positive | HPV-negative |
| --- | --- | --- | --- | --- |
|  |  | N = 51 | n = 24 | n = 27 |
| Gender | Male | 44 | 19 | 25 |
|  | Female | 7 | 5 | 2 |
| Age | < 60 | 17 | 9 | 8 |
|  | 60–80 | 33 | 15 | 18 |
|  | > 80 | 1 | 0 | 1 |
| T stage | 1–2 | 32 | 16 | 18 |
|  | 3–4 | 19 | 8 | 11 |
| N stage | 0 | 6 | 4 | 2 |
|  | 1–3 | 45 | 20 | 25 |
| M stage | 0 | 51 | 24 | 27 |
|  | 1 | 0 | 0 | 0 |
| Stage | I­–II | 35 | 19 | 16 |
|  | III–IV | 16 | 5 | 11 |
| Smoking history | Never | 6 | 5 | 1 |
|  | Ever | 45 | 19 | 26 |
| Drinking habit | Yes | 43 | 18 | 25 |
|  | No | 8 | 6 | 2 |

**Supplementary Table 2.** A complete list of antibodies and conditions used for staining.

|  | Cycle 1 | Cycle 2 | Cycle 3 | Cycle 4 | Cycle 5 | Cycle 6 | Cycle 7 |
| --- | --- | --- | --- | --- | --- | --- | --- |
| Primary Ab | Hematoxylin | PD1 | CD4 | CD8 | CD3 | Foxp3 | Pan-CK |
| Clone/ Product |  | NAT105 | SP35 | SP7 | C8/144B | 236A/E7 | AE1/AE3 |
| Vender | Dako | Abcam | Abcam | Thermo Scientific | Thermo Scientific | eBioscience | Abcam |
| Conc | Original | 1/10 | Original | 1/100 | 1/50 | 1/40 | 1/2000 |
| Reaction | RT, 2 min | RT, 30 min | RT, 60 min | RT, 30 min | RT, 30 min | RT, 30 min | RT, 30 min |
| Secondary Ab |  | Anti-mouse | Anti-rabbit | Anti-mouse | Anti-rabbit | Anti-mouse | Anti-mouse |
| Reaction |  | RT, 30 min | RT, 30 min | RT, 30 min | RT, 30 min | RT, 30 min | RT, 30 min |
| AEC reaction |  | 5 min | 5 min | 5 min | 5 min | 5 min | 5 min |
